# A Smart System for Continuous Sitting Posture Monitoring, Assessment, and Personalized Feedback

**DOI:** 10.3390/s25185610

**Published:** 2025-09-09

**Authors:** David Faith Odesola, Janusz Kulon, Shiny Verghese, Adam Partlow, Colin Gibson

**Affiliations:** 1 Faculty of Computing, Engineering and Science, University of South Wales, Pontypridd CF37 1DL, UK; 30025293@students.southwales.ac.uk (D.F.O.); shiny.verghese@southwales.ac.uk (S.V.); 2 Rehabilitation Engineering Unit, Artificial Limb & Appliance Service, Cardiff and Vale University Health Board, Treforest Industrial Estate, Pontypridd CF37 5TF, UK; adam.partlow@wales.nhs.uk (A.P.); colin.gibson@wales.nhs.uk (C.G.)

**Keywords:** sitting posture classification, smart-sensing chair, machine learning, posture monitoring

## Abstract

Prolonged sitting and the adoption of unhealthy sitting postures have been a common issue generally seen among many adults and the working population in recent years. This alone has contributed to the alarming rise of various health issues, such as musculoskeletal disorders and a range of long-term health conditions. Hence, this study proposes the development of a novel smart-sensing chair system designed to analyze and provide actionable insights to help encourage better postural habits and promote well-being. The proposed system was equipped with two 32 × 32 pressure sensor mats, which were integrated into an office chair to facilitate the collection of postural data. Unlike traditional approaches that rely on generalized datasets collected from multiple healthy participants to train machine learning models, this study adopts a user-tailored methodology—collecting data from a single individual to account for their unique physiological characteristics and musculoskeletal conditions. The dataset was trained using five different machine learning models—Decision Tree (DT), Random Forest (RF), Support Vector Machine (SVM), K-Nearest Neighbors (KNN), and Convolutional Neural Networks (CNN)—to classify 19 distinct sitting postures. Overall, CNN achieved the highest accuracy, with 98.29%. To facilitate user engagement and support long-term behavior change, we developed SitWell—an intelligent postural feedback platform comprising both mobile and web applications. The platform’s core features include sitting posture classification, posture duration analytics, and sitting quality assessment. Additionally, the platform integrates OpenAI’s GPT-4o Large Language Model (LLM) to deliver personalized insights and recommendations based on users’ historical posture data.

## 1. Introduction

### 1.1. Background and Motivation

In recent years, sedentary behaviors such as prolonged sitting have become a fundamental part of many people’s lifestyle, especially office workers. Most find themselves confined to a desk in front of a computer screen for an extended period, a pattern that has proven detrimental to their health [1,2]. According to the World Health Organization (WHO), the economic burdens attributed to sedentary behaviors cost an estimated US $27 billion annually and are expected to reach US $300 billion by the year 2030 [3].

Additionally, the frequent adoption of unhealthy sitting postures, such as slouching and asymmetric sitting, is a contributing factor that increases the risk of several health issues, ultimately negatively impacting life expectancy. Unhealthy posture is not just prevalent among the older population but also across individuals within different age groups [4]. Exhibiting such a pattern over an extended period could lead to the development of long-term health issues such as lower back pains [5], scoliosis [6], and other musculoskeletal disorders [7]. Hence, it is typically advised by healthcare professionals to maintain a consistently symmetrical sitting posture, which includes keeping your back in a straight position, perpendicular to the chair’s backrest. Additionally, it is also recommended to avoid sitting for a prolonged period, especially if keeping the same position [8,9]. A few walking breaks are recommended within certain periods of the day.

To help combat the tendency towards unhealthy sitting and promote healthy posture, various researchers have proposed the use of smart-sensing chair systems that can detect various sitting postures. Currently, various methods are being employed to develop such systems, ranging from different classification methods, sensor types, sensor placement strategies, and feedback mechanisms. There is no doubt that sitting is a continuous task that often changes and varies among individuals of different bodily characteristics. Hence, the incorporation of corrective feedback systems is crucial in both promoting and recommending helpful sitting habits on a per-user basis. A review study by our team [10] highlighted a notable gap in the current research landscape—most studies primarily focus on classifying different sitting postures and achieving high classification accuracies, while giving minimal attention to the development of an effective feedback mechanism. The integration of a comprehensive feedback system that provides both informative analysis of a user’s sitting behaviors as well as actionable insights has the potential to yield positive behavioral change and improve overall user outcomes.

Traditionally, the development of posture recognition systems has relied on training machine learning models on datasets collected from multiple healthy volunteers. These datasets help build a highly generalizable machine learning model capable of recognizing many different sitting postures. While this approach can be effective for basic sitting posture classification tasks, such models do not consider an individual’s unique musculoskeletal structure and ergonomic setting, potentially leading to misclassification. The adoption of a user-centric approach could further enhance the accuracy and relevance of posture analysis by customizing the machine learning model to fit each person’s sitting pattern.

### 1.2. Research Objectives

The primary objective of this study is to design and implement a smart-sensing chair system fitted with two high-resolution pressure sensor arrays, capable of accurately classifying 19 distinct sitting postures using machine learning techniques and adopting a user-tailored approach. To evaluate the system’s performance, five machine learning algorithms were implemented and compared: Decision Tree (DT), Random Forest (RF), Support Vector Machine (SVM), K-Nearest Neighbors (KNN), and Convolutional Neural Networks (CNN). This paper also aims to develop a comprehensive posture feedback system that provides informative and personalized insights on one’s sitting habits.

## 2. Related Works

### 2.1. Sensors

In recent years, there has been a steady increase in research focused on sitting posture detection and monitoring systems. This growing interest underscores the potential of such technologies to significantly influence individual postural habits and, by extension, improve overall quality of life [10]. Currently, there are two main categories of posture monitoring systems: wearable devices and non-wearable devices. Wearable devices are systems fitted with sensors that must always be worn to capture real-time postural readings, most of which focus on the spinal area. These sensors are typically small and are often integrated into clothing that must remain in direct contact with the user’s body. As a result, even though they are small, many individuals may find them uncomfortable and potentially disruptive to their daily routines. In terms of the practicality of such systems among the general public, there is a great need to consider key areas such as energy consumption, portability, and the degree of obtrusiveness. For example, Pereira et al. [11] developed the Postural SmartVest, a wearable vest that utilized the smartphone’s built-in accelerometers to monitor the sagittal and coronal planes, while also offering multimodal (visual, auditory, and tactile) feedback for elderly users. Similarly, Hou et al. [12] proposed a smart garment embedded with textile strain sensors and vibration motors, aimed at detecting postural deviations and providing haptic feedback.

On the other hand, there are non-wearable solutions that do not require an individual to wear any special clothing or device. Most of these systems are unobtrusive by nature and capture postural measurements without being overly disruptive to the end user. Within this category, various methods can be employed to classify different sitting postures, including the use of image-based systems (e.g., video or depth cameras) and pressure-sensitive mats embedded with sensors, such as Force-Sensitive Resistors (FSRs) or load cells.

Image-based systems are designed to have a set of cameras that have been programmed to capture and track multiple reference points of the human skeletal joints, such as the neck, shoulders, and hips, to detect different sitting postures [13]. For image-based systems to function effectively, an unobstructed line of sight to the subject and proper lighting conditions are essential. Moreover, these systems can be quite intrusive and raise a significant concern regarding one’s privacy due to their continuous visual monitoring. Furthermore, their effectiveness can be mainly limited to controlled environments, making them an unpopular option among research studies [14]. 

The adoption of sensor-infused sitting mats is a widely used implementation, based on the notion of integrating one or more sensors into the backrest and seating area of a typical office chair. As the user engages in micro-movements while seated, subtle shifts and variations in the pressure distribution are detected by the integrated sensors. These pressure patterns are then used to train machine learning models to classify the different postures being adopted. Some of the commonly used sensors are pressure sensors, load cells, and flex sensors. One of the first research papers published that pioneered the idea of a smart-sensing chair system was by Tan et al. [15], back in 2001. They were able to classify 14 different sitting postures using a Principal Component Analysis (PCA)-based algorithm, which interfaced with a pressure sensor array module placed on both the backrest and the sitting area of the chair, achieving an overall accuracy ranging between 79% and 96%. Subsequently, many research studies have been published following a similar approach. Wang et al. [16] developed a smart chair system equipped with a (9 × 9) and (10 × 9) FSR pressure matrix, which were used to classify up to 15 different sitting postures using Spike Neural Networks (SNN). Tsai et al. [17] adopted a similar approach by using a textile-based pressure sensor array to classify seven sitting postures while achieving an overall classification accuracy of 85.90%.

### 2.2. Machine Learning Algorithms

The classification algorithm used to detect and distinguish between multiple sitting postures is a critical determinant of a smart-sensing chair system’s overall effectiveness. A wide range of approaches have been adopted in prior posture classification research, from simple rule-based techniques to statistical models and more complex deep learning architectures. These include algorithms such as K-Nearest Neighbors (KNN) [18,19], Decision Trees (DT) [20], Support Vector Machines (SVM) [17,21], Random Forests (RF) [22], Convolutional Neural Networks (CNN) [23], and Artificial Neural Networks (ANN) [24,25].

For this study, the primary focus was on comparing five representative classification algorithms: Decision Trees, Random Forests, Support Vector Machines, K-Nearest Neighbors, and Convolutional Neural Networks. These algorithms were selected to provide a diverse performance benchmark, covering models with varying complexity, interpretability, computational cost, and suitability for both structured and spatial data.

#### 2.2.1. Decision Tree (DT)

A decision tree is a supervised machine learning method that uses a flowchart-like, hierarchical structure to make predictions based on learned decision rules. It consists of a root node (starting point), internal nodes (which represent conditions or features), and leaf nodes (which denote outcome classes). Its intuitive interpretability and low training cost make it ideal for baseline comparisons. However, decision trees are prone to overfitting, especially as the tree becomes deeper and more complex, which can affect their generalization performance [26].

#### 2.2.2. Random Forest (RF)

Random Forest is a machine-learning model commonly used for regression and classification-type predictions. The random forest algorithm is composed of multiple decision trees that work together as an ensemble to output a final prediction. Each decision tree in the ensemble is trained on a random data subset, which is retrieved from a training sample using a method called bagging or bootstrap aggregating. This method introduces randomness into the data, reducing susceptibility to bias and overfitting across the trees. The random forest algorithm has three hyperparameters that must be considered: the node size, the number of trees, and the number of features. The final prediction is made by aggregating the outputs of all decision trees. Majority voting is typically used for classification-type predictions and group averaging for regression-type predictions [27,28]. Random Forest’s resilience to noise and ability to handle large feature spaces make it a widely used and reliable classifier in posture recognition tasks. 

#### 2.2.3. Support Vector Machine (SVM)

Support Vector Machine is a supervised machine learning model that finds the optimal hyperplane or dividing line that determines the boundaries between multiple data points based on defined classes. SVM aims to calculate the most optimal margin, which is defined as the distance between the hyperplane and the closest data points between multiple classes. For more complex and non-linear datasets, SVM uses kernel functions that perform a series of data transformations into a higher-dimensional space, making it easier to separate the data points into distinct classes [29]. This makes SVM especially useful in posture classification tasks involving subtle differences in pressure distributions.

#### 2.2.4. K-Nearest Neighbor (KNN)

K-Nearest Neighbor is another supervised machine learning technique that is used to solve classification and regression problems. The KNN algorithm works on the principle of making predictions based on the distance metric of a data point to the closest training data point. This means that the proximity between a data point and the K’s closest neighbor greatly determines its predictions. Unlike other models, KNN stores the training dataset in memory and performs all necessary computations during the prediction phase. While simple and intuitive, KNN can struggle with scalability and noise sensitivity in large datasets [30]. Nonetheless, its performance in many posture detection studies justifies its inclusion. 

#### 2.2.5. Convolutional Neural Networks (CNN)

The Convolutional Neural Network is a supervised neural network comprising multiple node layers: an input layer, one or more hidden layers, and an output layer. Each node across different layers is interconnected with one another and has an assigned weight and threshold value. The convolution layer is an essential layer of a CNN as it extracts relevant patterns or features by applying filters (convolution kernels), which makes it superior to other machine learning algorithms [31]. Its ability to automatically learn spatial hierarchies of features through convolution filters gives CNNs a significant advantage in posture classification, where body pressure patterns across a seat and backrest resemble structured 2D input data.

### 2.3. Feedback Mechanisms

A well-constructed feedback mechanism is primarily aimed at informing and encouraging an individual to adopt “healthier” sitting postures and providing valuable postural insights that would improve their overall sitting habits. According to existing studies, there are multiple ways that a user can receive useful feedback. Mobile phones have emerged as a popular medium for collecting and displaying helpful feedback to the end user. Cai et al. [32] developed a smart-sensing chair system that relayed the detected posture via a mobile app. Similarly, Cho et al. [33] developed a mobile app that provided statistical insights along with recommended relevant YouTube videos largely based on the sitting postures being adopted. Ran et al. [24] and Bourahmoune et al. [22] took a different approach, integrating haptic motors into the seating cushion, which vibrated whenever an improper sitting posture was detected and continued to vibrate until an upright posture was achieved. Ren et al. [34] incorporated an RGB LED light strip, which changed in color whenever the individual needed to change their sitting posture and take microbreaks.

### 2.4. Research Gap and Contributions

Over the years, significant progress has been made in the development of posture monitoring systems. However, a review of existing literature reveals that a majority of existing studies primarily focus on classifying multiple types of sitting postures with high accuracy using various machine learning algorithms. At the same time, limited attention has been devoted to the implementation of an effective and practical feedback mechanism that goes beyond mere posture recognition. Ideally, such a feedback system should encourage the end-user to adopt and maintain healthier sitting habits by providing relevant information and actionable insights to improve one’s well-being. Furthermore, this paper aims to fill this research gap by developing a comprehensive postural feedback system, the “SitWell” platform, consisting of both a web dashboard and a mobile application. These platforms aim to provide essential information regarding one’s sitting habits along with tailored recommendations using OpenAI’s GPT-4o Large Language Model (LLM). Additionally, this study contributes to the field by adopting a novel, user-centric approach in which the classification model is trained on data tailored to an individual’s unique musculoskeletal characteristics, thereby enhancing both personalization and relevance. 

## 3. Methods and Design

### 3.1. Design Requirements and System Architecture

**Figure 1:**
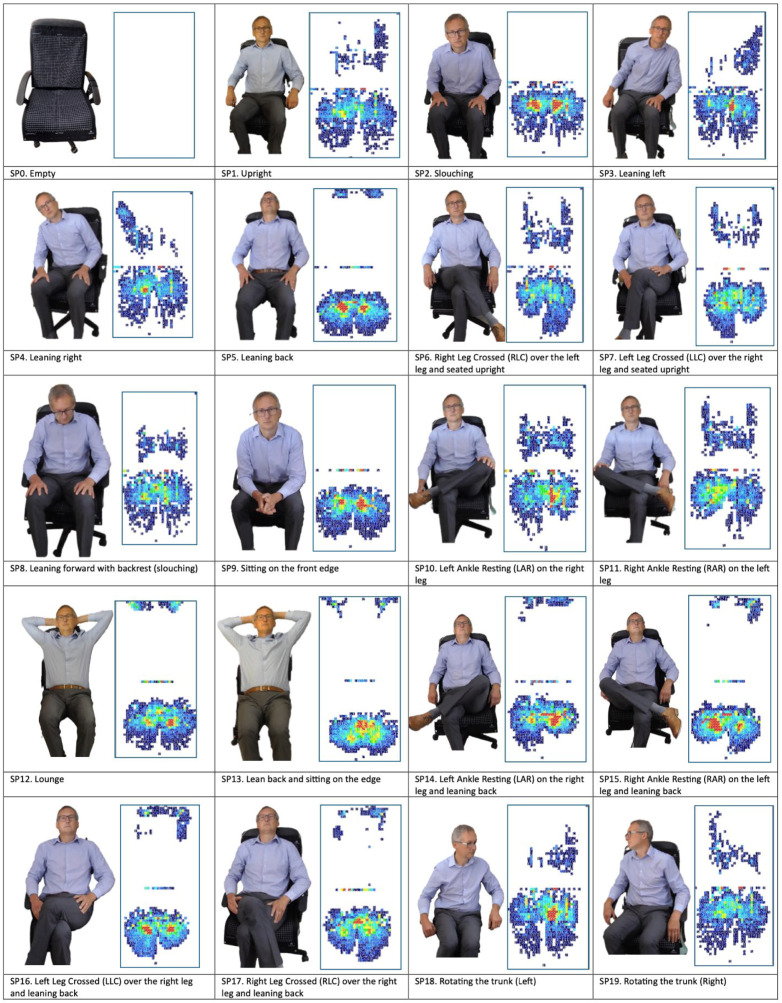
The 19 variations of sitting postures. (SP0) Empty, (SP1) Upright, (SP2) Slouching, (SP3) Leaning Left, (SP4) Leaning Right, (SP5) Leaning Back, (SP6) Right Leg Crossed (RLC) over the left leg and seated upright, (SP7) Left Leg Crossed (LLC) over the right leg and seated upright, (SP8) Leaning forward with a backrest (slouching), (SP9) Sitting on the front edge, (SP10) Left Ankle Resting (LAR) on the right leg, (SP11) Right Ankle Resting (RAR) on the left leg, (SP12) Lounge, (SP13) Lean back and sitting on the edge, (SP14) Left Ankle Resting (LAR) on the right leg and leaning back, (SP15) Right Ankle Resting (RAR) on the left leg and leaning back, (SP16) Left Leg Crossed (LLC) over the right leg and leaning back, (SP17) Right Leg Crossed (RLC) over the right leg and leaning back, (SP18) Rotating the trunk (Left), and (SP19) Rotating the trunk (Right).

This paper proposes the development of a smart-sensing chair capable of classifying 19 distinct sitting postures using two 32 × 32 pressure sensor arrays. All 19 sitting postures are listed in Figure 1.

**Figure 2:**
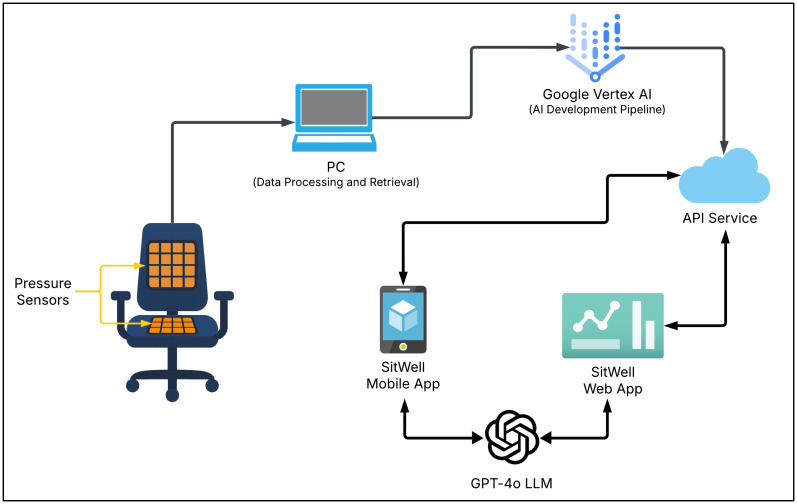
The entire system model of the proposed smart-sensing chair system.

The entire conceptual framework of the smart chair system is illustrated in Figure 2. The system comprises a standard office chair equipped with two pressure mats, one positioned on the backrest and the other on the seating cushion surface. This dual-sensor configuration enables comprehensive capture of the user’s posture by collecting pressure distribution data from both the upper and lower body. A personal computer (PC) was used to process and collect the sensor data readings and the associated metadata. After preprocessing steps such as normalization, data cleaning, and formatting were applied, the dataset was used to train machine learning models on Google Vertex AI, a fully managed cloud-based platform for building and deploying AI applications. Once the machine learning model had been both trained and evaluated, it was deployed as an Application Programming Interface (API) hosted within a cloud-based environment, enabling seamless integration with the “SitWell” platforms, which consist of a web-based dashboard and mobile application interface. Additionally, OpenAI’s GPT-4o Large Language Model (LLM) was integrated to deliver personalized insights and recommendations based on historical postural data.

### 3.2. Hardware Design

The CONFORMat system, developed by Tekscan Inc. (Norwood, MA, USA) [35], was selected for this smart-sensing chair application due to its high resolution and its intended use for biomedical research applications. Each sensor mat comprises 1024 independent pressure units (32 × 32) distributed over a 471.4 mm × 471.4 mm area. The value for each pressure unit ranges between 0 and 255. Additionally, each sensor mat is integrated with its data acquisition module, known as the Evolution handle device, which facilitates data transfer between the sensor array and a PC via a tethered USB cable, with sampling rates of up to 100 Hz. A summarized technical specification list of the CONFORMat system is provided in Table 1.

### 3.3. Data Collection

#### 3.3.1. Experimentation Setup

**Figure 3:**
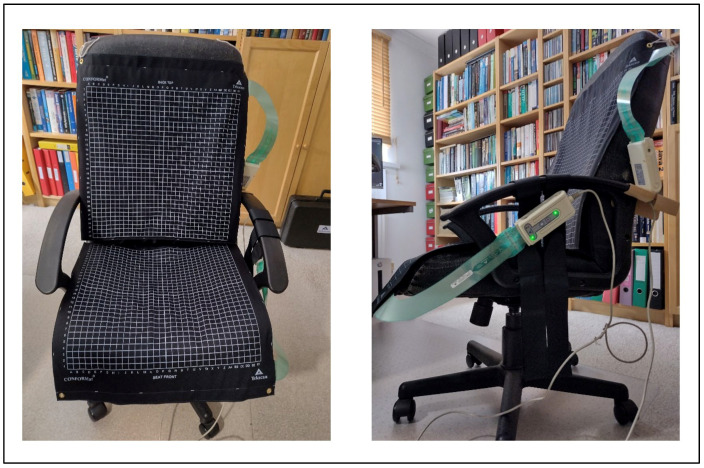
An office chair equipped with two Tekscan CONFORMat Pressure Sensor Mats.

For the experiment, two CONFORMat pressure sensor arrays were used: one on the backrest and the other on the sitting cushion, as shown in Figure 3. The integration of both pressure sensor mats enabled the comprehensive capture of the individual’s full spatial sitting data. The thin and flexible nature of the pressure sensor mats allowed for seamless integration into the chair’s surfaces without compromising sitting ergonomics or user comfort.

#### 3.3.2. User-Centric Approach in Data Collection

In contrast to similar studies, this study adopted a user-centric approach by collecting the sensor dataset and training a machine learning model tailored to a single individual. It has been acknowledged that other studies typically adopt the traditional approach of involving a diverse set of healthy volunteers in the data collection stage, which helps develop a highly generalized model aimed at universal use. However, the fundamental flaw with this methodology lies in its heavy reliance on the assumption that postural patterns are universally consistent across all users, while overlooking the fact that individual characteristics—such as skeletal structure, muscle composition, and medical conditions—often play a significant role in shaping unique or unconventional sitting habits. For instance, individuals with musculoskeletal disorders or those who use a wheelchair regularly may have developed unique postural habits that do not align with conventional definitions of “healthy” posture. A generalized model may misclassify their most comfortable or ideal sitting posture as unhealthy, making the system ineffective or even misleading [14]. Nadeem et al. [37] also pointed out that developing a well-generalized machine learning model can be a complex task due to the variability in body types and sitting behavior among different individuals; they therefore recommend using a more personalized approach. Moreover, ergonomic factors such as chair design—specifically backrest angle, seat height, armrest configuration, and overall geometry—can also affect pressure sensor readings, yet are often overlooked in related studies. To address this limitation, it is proposed that future versions of the mobile application include a brief calibration cycle, wherein the user is prompted to assume different postures for short periods. This would allow the system to retrain the model using the user’s own sensor data, effectively personalizing it to their unique anatomy and ergonomic environment. 

For the data collection phase in this study, a single participant was instructed to sit in 19 different postures, as previously highlighted in Figure 1. Each posture was held for approximately 25 s, during which pressure data was continuously recorded and saved. A total of 151 frames of sensor data were captured for each given posture, resulting in 2869 sets of data overall. After data collection, a set of pre-processing steps was performed, which involved data labeling, normalizing the dataset using the min-max normalization technique, and removing empty data frames.

### 3.4. Development of a Personalized Machine Learning Model 

**Figure 4:**
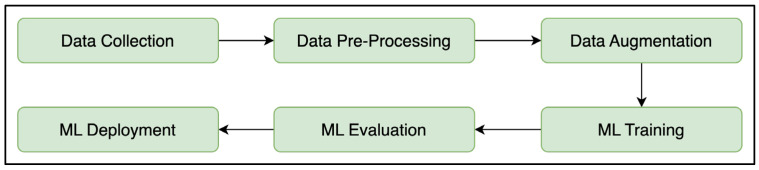
The 6-stage Machine Learning Development Pipeline.

The development of a personalized machine learning model tailored to a particular individual requires the establishment of a systematic pipeline that ensures consistent data flow and model quality. The proposed 6-stage pipeline, illustrated in Figure 4, provides a framework for the steps involved in such an approach.

The first stage is the user data collection session, during which the individual is instructed to adopt different sitting postures over a short period. The next stage is the data pre-processing stage, where the dataset is labelled and cleaned before proceeding to further steps. The next step is data augmentation, where the dataset would be synthetically augmented to further improve the machine learning model’s robustness. The machine learning model would then be trained using the augmented dataset and evaluated before it is finally deployed.

#### 3.4.1. Data Augmentation

**Figure 5:**
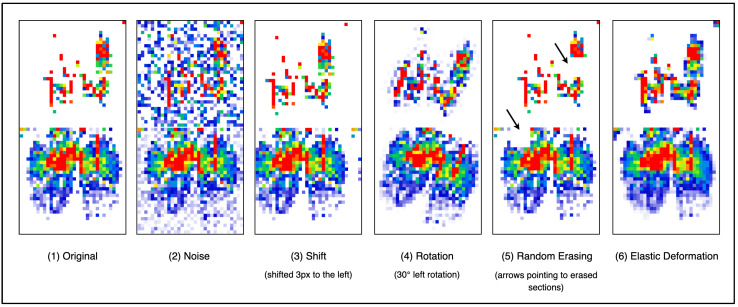
A series of examples of data augmentation being used. (1) Original—an upright posture without transformation. (2) Noise—an upright posture with added noise. (3) Shift—an upright posture shifted to the left. (4) Rotation—an upright posture rotated counterclockwise. (5) Random Erasing—randomly erases certain sections of the upright posture data. (6) Elastic Deformation—elastically smoothens the upright posture data.

Given that there was only one participant involved in the data collection phase, there was a need to augment the dataset to further improve the machine learning model’s performance and reduce the risk of overfitting. Hence, to accomplish this, the dataset was synthetically augmented by applying a random combination of predefined transformations: noise, shift, rotation, random erasing, and elastic deformation. Figure 5 illustrates the effect of each transformation applied to the upright sitting posture data, along with the parameters for each transformation listed in Table 2.

The noise transformation was designed to replicate a real-world scenario in which environmental factors, such as temperature, electromagnetic interference, humidity, and user clothing, could negatively impact sensor readings. The shift transformation accounts for a scenario where individuals are not perfectly seated in the center of the seat’s cushion or when the sensors have been slightly shifted from their center position. The rotational transformation considers periods where the user might be maintaining a particular posture but in a slightly rotated manner, or if the sensors were to be slightly rotated off their intended position, which might be a common issue. The random erasing transformation arbitrarily removes certain sections of the sensor reading, replicating certain areas of the sensor that are faulty or partially blocked. Finally, the elastic deformation slightly distorts the sensor readings to account for situations where there are minimal variations in a user’s body structure over time.

Hence, by applying these transformation techniques, the variability of the dataset increased compared to the original dataset. Additionally, during the data collection stage, slight posture adjustments were made to intentionally simulate natural variability. Overall, as a result, the entire dataset increased from 2302 to 8607 data frames.

#### 3.4.2. CNN Architecture for Posture Classification

**Figure 6:**
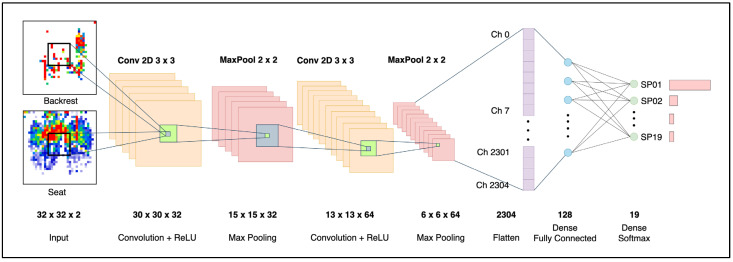
The design of the proposed CNN architecture.

The proposed CNN architecture processes dual 32 × 32 input matrices to classify sitting postures across 19 distinct categories, with the detailed network structure illustrated in Figure 6. The CNN architecture begins with the first convolutional layer, which is equipped with 32 filters that extract spatial features using the ReLU activation function. The Max Pooling Layer then followed, reducing the spatial resolution while retaining the relevant key activations and further reducing the risk of overfitting. The following second Convolutional Layer was equipped with 64 filters to further learn and make sense of the spatial pattern that was processed by the previous layer. A second Max Pooling Layer was added to reduce the data’s dimensionality further and extract the essential features. The Flatten Layer then transformed the two-dimensional feature map to a one-dimensional vector, which was required by the subsequent two dense layers. The first Dense Layer consisted of 128 neurons, along with the ReLU activation function, and the second Dense Layer was the final output layer, which comprised 19 neurons corresponding to the 19 postures being classified. The CNN model was trained using the Adam optimizer to fine-tune the learning rates, along with early stopping and the cross-entropy loss to measure the performance. Overall, this CNN architecture leverages a lightweight and shallow network layer topology, striking a balance between its feature extraction capability of identifying 19 different postures and computational efficiency, making it ideal for real-time and personalized use.

#### 3.4.3. Other Machine Learning Algorithms for Posture Classification

A series of hyperparameter tuning experiments was conducted on the other classification algorithms to determine the optimal settings that would yield the highest accuracy. The Grid Search technique was used to determine the optimal sets of parameters for these classifiers, with accuracy serving as the scoring metric. The DT algorithm was evaluated at depths of 5, 10, 20, and 30 to assess the likelihood of overfitting. For the RF algorithm, the number of estimators (n_estimators) was varied around 50, 100, and 200, along with tree depths (max_depth) at around 10, 20, and 30. For the SVM, we explored with different regularization parameter C value ranges such as 0.1, 1, and 10. For the KNN classifier, several neighbor values were tested, including 3, 5, 7, and 9, to achieve the best balance between bias and variance. The 5-fold cross-validation was used to evaluate the performance of the KNN across this list of values.

#### 3.4.4. Training and Validation Approach 

The dataset was divided into three subsets to train, fine-tune, and evaluate each machine learning algorithm: 80% for training, 10% for validation, and the remaining 10% for testing model performance. After training the model, the results were analyzed using the confusion matrix, which assesses the accuracy of each model by comparing the predicted value with the true value over a series of key criteria, which are as follows: True Positives (TPs), True Negatives (TNs), False Positives (FPs), and False Negatives (FNs). Additionally, several performance metrics were calculated, including accuracy, precision, recall, and the F1-score values. The accuracy represents the percentage with which it accurately identified the different postures across the entire testing dataset. Precision is a classification metric that measures the proportion of correctly predicted positive instances out of all instances predicted as positive. It reflects how accurately the algorithm identifies the predicted postures. The recall value reflects the number of times the machine learning algorithm correctly identified an actual posture. Meanwhile, the F1-score metric provides an overall assessment that considers both precision and recall values.

### 3.5. Postural Feedback Techniques

#### 3.5.1. Personalized Feedback Using LLM

**Figure 7:**
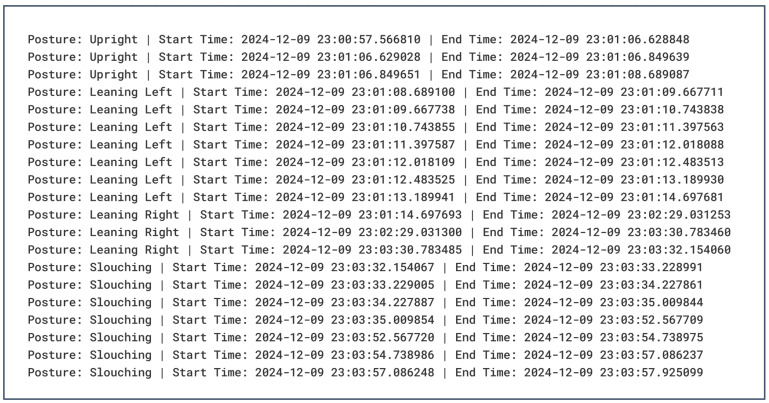
A sample historical postural dataset along with its timestamp.

To generate personalized and tailored recommendations for the end user, OpenAI’s Generative Pre-Trained Transformer (GPT) model was leveraged as the core component. GPT is an LLM that has been pre-trained on a vast amount of publicly available data, making it very useful in many areas, such as data analysis, language learning, pattern matching, and sentiment analysis, to name a few [38]. OpenAI has a variety of models available; for this research study, the GPT-4o model was selected due to its status as one of their most capable and flagship models available at the time. The model parameters were fine-tuned using the following settings shown in Table 3. Furthermore, the model was provided with a realistic set of an individual’s historical sitting postural dataset and a system prompt that instructed it to identify recurring postural issues and recommend better postural habits to improve one’s health and well-being. A sample of the historical dataset being passed to the model can be seen in Figure 7. Each data entry contains the detected sitting posture and the corresponding start and end timestamps.

#### 3.5.2. Postural Assessment Using Borg CR-10 Scale

The Borg CR-10 Scale, originally designed to quantify perceived exertion (e.g., fatigue, effort) on a scale from 0 (“nothing at all”) to 10 (“maximal effort”), is a validated psychophysical tool for subjective assessment of physical strain during activity [39,40]. In this study, the scale was adapted to evaluate biomechanical risk rather than exertion, with scores categorized as follows: 1–3 (optimal), indicating minimal risk to musculoskeletal, circulatory, or neurological systems; 4–6 (moderate), reflecting transient stress resolvable through posture modification; and 7–10 (harmful), denoting postures with elevated risks of chronic injury or dysfunction. This adaptation is grounded in methodologies from ergonomic and biomechanical literature, which correlate posture-related harm with objective metrics such as spinal disc pressure [41], muscle activation patterns [42], and impaired blood flow [43].

While the proposed scores are derived from a systematic synthesis of existing evidence, they are inherently provisional. The framework allows for adjustment based on emerging data or individual musculoskeletal health status (e.g., pre-existing conditions, variability in pain perception). Consequently, the scoring thresholds are presented as a flexible guideline rather than a definitive classification, as shown in Table 4.

#### 3.5.3. Sitting Posture Quality Assessment

Given that the prolonged adoption of static and unhealthy sitting postures is a significant contributor to musculoskeletal disorders, a dynamic posture scoring mechanism was developed which considers both the posture being adopted and its duration. The posture scoring metric (QS) is computed using the formula as defined:

(1) $$QS=\frac{1}{\left(1+S+D*T\right)}$$

where S denoted the associated Borg CR-10 Score, T represents the amount of time (in minutes) the posture was maintained, and D is the decay factor that influences the penalty applied based on the postural context and duration, defined in Table 5. For upright postures, a threshold limit of 30 min was implemented, beyond which a decay factor value is applied. This aligns with the fact that maintaining static postures for an extended period significantly increases the risk of musculoskeletal disorders. Upright postures, although often considered ideal, can also be detrimental and increase the risk of lower back pain when maintained for an extended period. To further support this, other studies [55,56] have recommended avoiding maintaining a static posture for longer than 20 to 30 min at a time or adopting a dynamic sitting pattern, which involves alternating between different postures. 

Additionally, the delay factor values, which are editable, were formulated through a series of tests that best reflect the cumulative impact of both postural context and duration. For non-upright postures maintained for a short duration, a tolerance threshold of 30 s was established, allowing for brief periods of postural adjustment. Meanwhile, maintaining non-upright postures beyond this threshold results in higher decay factor values. 

### 3.6. SitWell Feedback Platform

The “SitWell” platform, consisting of both a web dashboard and mobile application, was developed to provide relevant information about one’s sitting habits. The web and mobile application were built using Flutter (version 3.22.1), an open-source framework that supports the development of multi-platform applications, such as iOS, Android, and Desktop, under a single codebase. Additionally, a serverless backend application using the Python programming language (version 3.12) was developed and integrated with the pre-trained CNN model for posture prediction. Finally, OpenAI’s GPT-4o LLM was then integrated to generate personalized recommendations based on historical postural data. 

**Figure 8:**
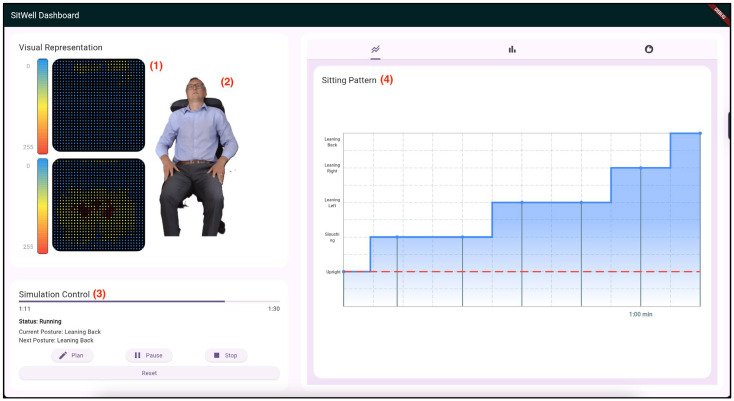
First screenshot of the “SitWell” Dashboard. (1) Pressure Sensor Mat Visualization; (2) A demonstration of the sitting posture being adopted; (3) The Simulation Control Panel; (4) A line chart visualizing the different sitting postures being adopted over time.

**Figure 9:**
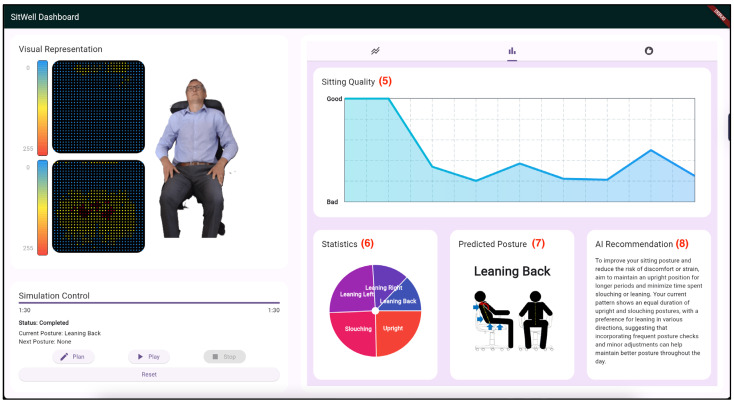
Second screenshot of the “SitWell” Dashboard. (5) A line chart showing the quality of the sitting over time; (6) A Pie chart displaying the statistics of the sitting posture being adopted; (7) The predicted posture based on the pre-trained CNN model; (8) A summarized recommendation generated by the GPT-4o LLM based on historical data.

**Figure 10:**
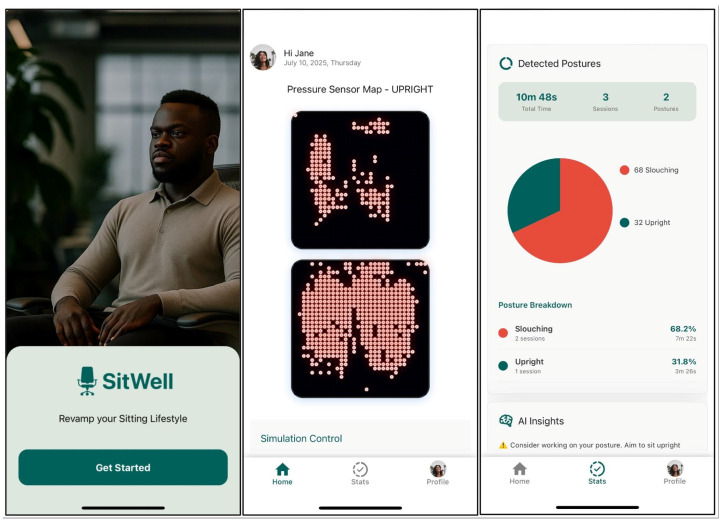
Mobile Application screenshots. The first screenshot is the ‘Get Started’ screen, the second screenshot is the ‘Home’ screen, which displays a visualization of the detected posture, and the third screenshot is the ‘Statistics’ screen, showing all relevant information regarding the user’s postural habits.

Due to technical limitations, the feedback application doesn’t retrieve real-time data from the pressure sensor mats. Instead, the previously collected postural sensor dataset was programmatically integrated and fed into the posture feedback system to replicate it coming directly from the sensor mat through wireless connectivity. Figure 8, Figure 9 and Figure 10 are screenshots of the web and mobile applications that were developed.

## 4. Results and Discussion

### 4.1. Performance of the Machine Learning Algorithms

The data revealed that the CNN yielded the best results and significantly outperformed other statistical machine learning models. CNN achieved an accuracy of 98.29%, a 98.18% F1-score, 98.23% precision, and 98.14% recall. This was followed by KNN, which achieved 91.03% accuracy. It was closely followed by RF, which achieved an accuracy of 90.27%. The worst-performing machine learning algorithm was DT with 66.29%. The entire results are summarized in Table 6. 

### 4.2. The Performance Results for CNN

**Figure 11:**
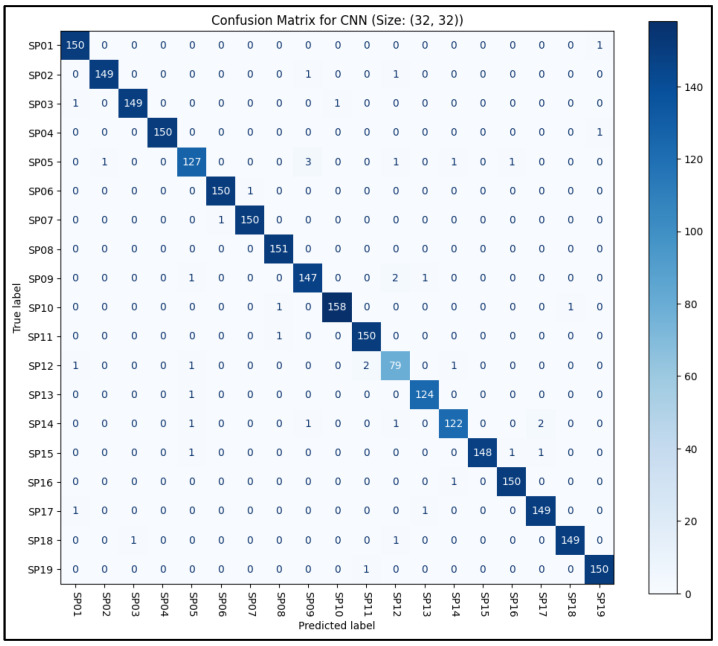
Confusion Matrix for the CNN model.

Given that the CNN model achieved the highest classification accuracy among all the machine learning models tested, its performance was further examined by analyzing the confusion matrix, as illustrated in Figure 11. The matrix demonstrated excellent performance, with the CNN accurately distinguishing between the 19 distinct sitting postures for the vast majority of the testing dataset. Notably, the confusion matrix revealed that the SP12 (lounge) posture class was significantly underrepresented. This underrepresentation was a consequence of the pre-processing phase, during which a number of empty or invalid data frames, particularly from this class, were removed, thereby reducing their overall presence in the dataset.

**Figure 12:**
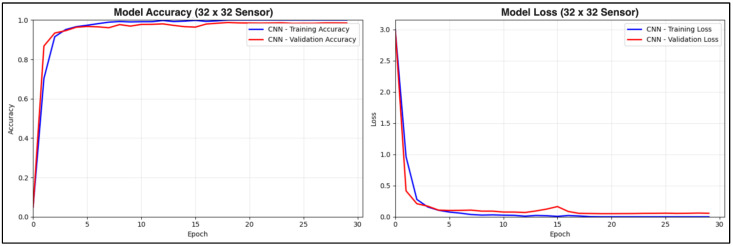
The training curves of the CNN model. The first graph illustrates the progression of CNN’s accuracy through each epoch. The second graph is the progression of the loss value across each epoch.

The learning curves in Figure 12 illustrate the convergence of training and validation accuracy to nearly 99% at approximately Epoch 23. Additionally, the close tracking of both training and validation metrics suggests that there are no issues with overfitting.

### 4.3. Comparison Between the Pressure Sensor Density and Machine Learning Model Accuracy

As previously discussed, the resolution of both the backrest and seat pressure sensor mats that were used was 32 × 32, each comprising 1024 pressure-sensing units. A resolution of this magnitude can be considered relatively excessive for widespread adoption, primarily due to the significant costs associated with such a system. Hence, there was a need to investigate whether a lower-resolution sensor could still achieve the same level of accuracy as the original 32 × 32 sensor array. To test this, a series of experiments was conducted by programmatically lowering the original (32 × 32) sensor resolution to multiple smaller simulated sensor resolutions (2 × 2, 3 × 3, 5 × 5, 10 × 10, 20 × 20, 32 × 32) using the bilinear interpolation algorithm across the entire dataset. These modified datasets were then trained on the predefined list of machine learning algorithms.

**Figure 13:**
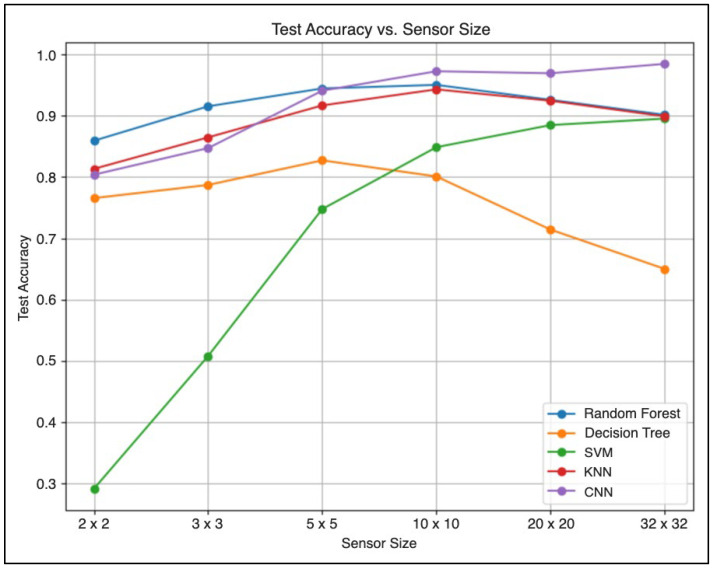
A graph comparing the accuracies of different machine learning models concerning varying sensor sizes.

Overall, as expected, the classification accuracy for each machine learning algorithm was strongly influenced by the sensor resolution, as shown in Figure 13. Most of the machine learning models struggled with lower resolutions, such as those of 2 × 2, 3 × 3, and 5 × 5 resolutions. This suggests that these resolutions do not provide sufficient information or feature sets for the machine learning models to reliably identify different sitting postures. As a result, this caused the models to perform relatively poorly compared to those of higher resolutions. Interestingly, it can be seen that the 10 × 10 array size is sufficient for most machine-learning models to distinguish between different sitting postures without a significant loss in accuracy. Notably, the CNN consistently outperformed all other models across varying resolutions, with its accuracy improving as resolution increased. On the other hand, a decline in performance was observed for the DT, RF, and KNN models at higher resolutions, indicating potential overfitting issues that may necessitate further hyperparameter tuning.

### 4.4. Cost-Effectiveness of Pressure Sensing Technologies

In terms of practicality, it is essential to assess the cost-effectiveness of commercially available pressure-mat systems similar to the one used in this study. High-fidelity, research-grade pressure mapping systems, such as those manufactured by Tekscan Inc. (Norwood, MA, USA) or XSensor (Calgary, AB, Canada), offer high resolution and accuracy. These systems are typically sold as integrated packages that include proprietary software and data acquisition hardware, which simplifies any experimental setup. However, the associated cost, with a single sensor mat ranging from approximately $3000 to over $8000 USD, renders them unsuitable for scalable deployment in consumer-grade applications. Their primary market is, therefore, well-funded research laboratories and clinical environments.

In contrast, systems built with low-cost, sparsely distributed sensors like Force-Sensitive Resistors (FSRs) offer a more viable alternative [14,17,25]. These technologies are widely available from electronic component distributors and are exceptionally affordable, with individual sensors often costing between $5 to $10 USD. While the low unit price is attractive, it comes with the trade-off of requiring significant investment in research and development, including custom hardware design and the creation of algorithms to process and interpret the raw sensor data. Despite this, existing studies have proven that a limited number of strategically placed FSRs can effectively classify multiple sitting postures without needing a high-resolution grid [17].

### 4.5. Evaluation of AI Recommendation

**Figure 14:**
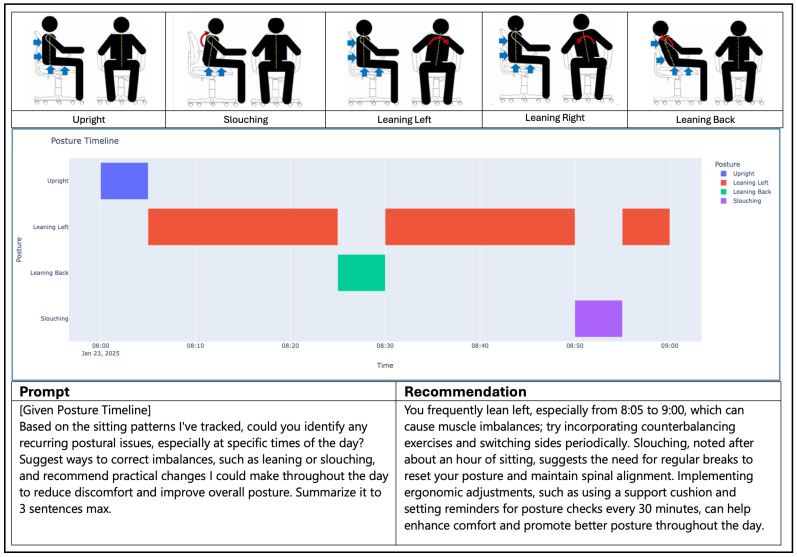
A simulated experiment of an individual’s posture timeline leaning left for an extended period, along with the LLM’s given prompt and generated recommendation.

The evaluation of the LLM’s generated recommendations was conducted by experimenting with varying system prompts. Once a satisfactory prompt structure was developed, a simple test environment was created to analyze the LLM’s ability to produce accurate, well-defined recommendations. In this environment, a pre-recorded scenario was simulated, representing an individual’s postural timeline over a one-hour period, during which the individual maintained various postures for extended durations, as illustrated in Figure 14. The prompt tab at the bottom left of the figure shows the prompt that was crafted for the LLM, and the recommendation tab to the right displays the results generated by the model. Overall, the model accurately highlighted the individual’s leaning left for an extended period. It also provided additional suggestions to improve the subject’s sitting habits. After conducting further tests like this, it was observed that the LLM’s responses were accurate enough to understand the sitting patterns that were adopted over a given period. However, it was noticeable that the responses generated were at times overly generic, suggesting areas for improvement in the prompt to make it more tailored to the individual.

## 5. Conclusions

In this paper, a smart-sensing chair system capable of classifying 19 sitting postures using two 32 × 32 pressure sensor mats was developed, for which a user-tailored methodology was adopted. The robustness of the model was enhanced by synthetically augmenting the dataset to reduce the risk of overfitting. Five different machine learning algorithms (DT, RF, SVM, KNN, and CNN) were also examined and compared. Overall, the highest classification accuracy of 98.29% was achieved by the CNN algorithm, while the lowest score of 66.29% was recorded for the DT. Furthermore, the impact of sensor resolution on performance was investigated, and it was found that a lower-resolution 10 × 10 array was sufficient for most models to achieve high accuracy, indicating a path toward more cost-effective hardware solutions.

Additionally, a comprehensive feedback system, comprising both web and mobile applications, was developed to provide the end user with valuable information regarding adopted sitting patterns. This platform is characterized by a dynamic posture scoring mechanism through which sitting quality is evaluated based on both the biomechanical risk of the posture and its duration, whereby static positions maintained for extended periods are penalized. Insightful recommendations and actionable plans are generated based on historical data using the OpenAI GPT-4o LLM.

## 6. Future Directions

While this study demonstrates the effective use of a high-resolution sensor system, such as the Tekscan CONFORMat, in accurately classifying multiple sitting postures, it would be valuable to explore more cost-effective sensor alternatives. Establishing a systematic methodology for determining the optimal placement of each sensor unit, especially in sparsely distributed configurations, remains a key area for future investigation. To aid this, future work could focus on the interpretability of the CNN model using methods such as Grad-CAM, SHAP (Shapley Additive exPlanation), or Local Interpretable Model-Agnostic Explanations (LIME). These techniques could visually highlight the most influential pressure-sensitive regions for classification, thereby providing a data-driven basis for optimizing sensor placement in low-cost designs. A scientific comparison between the development of a personalized machine learning model using a user-tailored approach and a generalized model pre-trained against a wide set of volunteers would be insightful for understanding the strengths and limitations of both approaches. 

Due to technical constraints, the current feedback system was unable to stream real-time posture data from the pressure sensors. Thus, future work should focus on implementing wireless data transmission using Internet of Things (IoT) protocols to enable seamless, real-time integration with the available feedback platforms. Integrating additional sensors, such as Electrocardiograms (ECGs) and other health sensors, could provide a comprehensive view of the user’s overall health and sitting lifestyle. User feedback on the “SitWell” platform will also be essential to refine usability and effectiveness. In addition, collaboration with healthcare professionals, particularly those in rehabilitation engineering centers, could help validate and enhance the medical relevance of the LLM-generated recommendations, ensuring that they are both actionable and clinically appropriate. Additionally, double-blind controlled clinical trials should be considered to evaluate the platform’s effectiveness.

## Figures and Tables

**Table 1:** CONFORMat sensor mat’s full technical specifications [36].

Technical Specification	Details
System Model	CER2 (CONFORMat Sensor)
Sensor Model	5330
Quantity	2
Sensing Area	471.4 mm × 471.4 mm (18.56 in. × 18.56 in.)
Number of Sensing Elements	2048 (1024 on each mat)
Pressure Range	0-34 kPa (5 psi)
Spatial Resolution	0.5 Sensel/cm^2^ (3.0 Sensels/in^2^)
Sampling Rate	Up to 100 Hz

**Table 2:** A summary of the transformation techniques that were used to augment the dataset.

Transformation	Parameters
Noise	Gaussian Noise (noise level = 0.5)
Shift	x-shift = ±10 pixels y-shift = ±10 pixels
Rotation	±30 degrees
Random Erasing	area = 10%
Elastic Deformation	Alpha = 24, sigma = 4

**Table 3:** OpenAI GPT-4o’s Parameters.

Parameter	Value
Model	GPT-4o
Temperature	1
Max Token	256
Top P	1
Friction Penalty	0
Presence Penalty	0

**Table 4:** Enhanced Posture Evaluation Matrix (1–10 scale; 1 = optimal, 10 = most harmful), synthesizing biomechanical risks across 19 sitting postures.

Posture	Score	Description	Scientific Justification
SP1	1	Neutral spine, lumbar support, 90° hip/knee angles	Highest quality of sitting: preserves natural lordosis, minimal muscle activation [44].
SP2	10	Unsupported forward trunk flexion	Worst posture: increases intradiscal pressure, resulting in a rapid onset of low-back pain and fatigue [44,45].
SP3	7	Lateral trunk flexion/weight shift left	Asymmetrical posture induces pelvic obliquity, scoliosis risk; quality of sitting compromised by uneven load [46,47].
SP4	7	Lateral trunk flexion/weight shift right	Mirror of SP3: similar pelvic tilt and muscle imbalance [46,47].
SP5	3	Semi-recline (10–130°) with lumbar support	Reduces the spinal disc pressure, improves comfort, and recommended for breaks [48].
SP6	8	Right leg crossed over left thigh, torso upright	Elevates one hip, lateral rotation; degrades sitting symmetry and lumbar alignment [49,50].
SP7	8	Left leg crossed over right thigh, torso upright	Mirror of SP6: similar pelvic torsion and musculoskeletal strain [49,50].
SP8	9	Trunk flexion despite back support	Backrest fails to preserve lordosis; high risk of chronic discomfort [44,51].
SP9	6	Buttocks at seat edge, anterior pelvic tilt	Opens hip angle, engages core; moderate quality but fatiguing over time [52].
SP10	7	Left ankle on right knee, torso upright	Asymmetric leg cross; reduces sitting quality and induces rotational shear [49,53].
SP11	7	Right ankle on left knee, torso upright	Mirror of SP10; similar lateral tilt and discomfort [49,53].
SP12	4	Full back support, legs forward	Simulates supine; lowest spinal load; good recovery posture but not for work tasks [54].
SP13	9	Perch edge with back lean, no lumbar support	Combined perch and recline; significant instability and posterior tilt [44].
SP14	10	Left ankle on right knee with unsupported recline	Worst combined posture: maximal torsion and flexion harm [49].
SP15	10	Right ankle on left knee with unsupported recline	Mirror of SP14: carrying identical risks [49].
SP16	10	Right leg crossed with unsupported recline	Compound cross with slouching: highest spinal stress [44].
SP17	10	Left leg crossed with unsupported recline	Mirror of SP16: same risk level [44].
SP18	8	Torso twisted left, hips fixed	Sustained rotation; increases facet-joint shear and disc stress [51].
SP19	8	Torso twisted right, hips fixed	Mirror of SP18: similar rotational load [51].

**Table 5:** Decay factor conditional rulesets.

ID	Postural Context	Duration Condition	Decay Factor
1	Upright	T ≤ 30 min	0.0
2	Upright	T > 30 min	0.005
3	Not Upright	T ≤ 30 s	0
4	Not Upright	T > 30 s	0.02

**Table 6:** The accuracy, F1-score, precision, and recall metrics of each of the machine learning algorithms.

Model	Parameters	Accuracy	F1-Score	Precision	Recall
DT	depth = 30	0.6629	0.6615	0.6652	0.6578
RF	depth = 30, n_estimator = 200	0.9027	0.9012	0.9078	0.8947
SVM	C = 0.1	0.8969	0.8949	0.8967	0.8931
KNN	k = 3 neighbors	0.9103	0.9113	0.9210	0.9020
CNN	epoch = 23	0.9829	0.9818	0.9823	0.9814

## Data Availability

Data will be available upon request.
